# Experimental and Simulation Investigation on Fatigue Performance of H13 Steel Tools in Friction Stir Welding of Aluminum Alloys

**DOI:** 10.3390/ma17071535

**Published:** 2024-03-28

**Authors:** Ling Long, Xiaohong Zhang, Song Gu, Xiuxin Li, Xuefeng Cheng, Gaoqiang Chen

**Affiliations:** 1 Aviation Equipment Manufacturing Industry College, Chengdu Aeronautic Polytechnic, Chengdu 610100, China; 2 Advanced Welding Research Center, Sichuan Aerospace-Changzheng Equipment Manufacturing Co., Ltd., Chengdu 610100, China; 3 Department of Mechanical Engineering, Tsinghua University, Beijing 100084, China

**Keywords:** friction stir welding, FSW ALE model, welding tool, fatigue performance

## Abstract

As the central component in friction stir welding, the design and manufacture of welding tools for aluminum alloys have garnered substantial attention. However, the understanding of tool reliability during the welding process, especially in terms of fatigue performance, remains unclear. This paper focuses on the welding of AA2219-T4 as a case study to elucidate the predominant failure mode of the tool during the friction stir welding (FSW) of aluminum alloys. Experimental methods, including FSW welding and fracture morphology analysis of the failed tool, coupled with numerical simulation, confirm that high-cycle mechanical fatigue fracture is the primary mode of the tool failure. Failures predominantly occur at the tool pin’s root and the shoulder end face with scroll concave grooves. The experimental and simulation results exhibit a noteworthy agreement, validating the reliability of the simulation model. The FSW Arbitrary Lagrangian–Eulerian (ALE) model developed in this study analyzes stress distribution and variation under the thermo-mechanical coupling effect of the tool. It reveals that stress concentration resulting from structural changes in the tool is the primary driver of fatigue crack initiation. This is attributed to exposure to alternating cyclic stresses such as bending, tension, and torsion at the tool pin’s root, manifesting as multiaxial composite mechanical fatigue. Among these stresses, bending alternating cyclic stress exerts the most significant influence. The paper employs the Tool Life module in DEFORM software to predict the fatigue life of the tool. Results indicate that reducing welding speed or increasing rotation speed can enhance the tool’s fatigue life to some extent. The methodology proposed in this paper serves as a valuable reference for optimizing FSW structures or processes to enhance the fatigue performance of welding tools.

## 1. Introduction

Friction stir welding (FSW) has rapidly advanced as a solid-state welding technology, employing a high-speed rotating stirring tool to generate heat through friction during contact with workpieces. This localized heating induces plastic flow in the material, leading to metallurgical bonding and the formation of dense welded joints. FSW is recognized for its simplicity, environmental friendliness, and reliability, particularly in welding materials like aluminum alloys, which are challenging to weld using traditional fusion welding methods. Therefore, FSW has been found to have extensive applications in various industrial sectors, notably in the aerospace, rail transportation, and maritime industries [1,2]. The welding tool, which is a pivotal component in the FSW process, not only influences welded joint quality due to its structure design and manufacturing but may also encounter wear, deformation, crack, and fatigue failures [3,4,5]. Thus, in addition to exploring the welding mechanisms of FSW, it is imperative to investigate the failure mechanisms of the welding tool during FSW and establish reliable methods for tool life prediction to mitigate economic losses caused by uncertainty in tool service life.

The failure modes of the welding tool in the FSW process vary due to differences in material properties and thermal load-bearing capacity [6,7]. Considering the outstanding mechanical properties, including high wear resistance, elevated temperature strength and toughness, and fatigue resistance, coupled with the added benefits of cost-effectiveness and ease of manufacturing, H13 tool steel stands out as the preferred material for producing welding tools employed in the FSW of aluminum alloys in the industry [8,9]. However, under different conditions, such as workpiece materials, welding process parameters, tool structure and dimensions in the FSW process, the welding tool may exhibit poor durability, which leads to various failures. Therefore, the research on the failure mechanisms of welding tools made of H13 steel materials in the FSW process has attracted widespread attention from scholars. Prado et al. [10] found no significant tool wear in the FSW of aluminum 6061 when experimentally measuring the rate of tool wear. However, Hasieber et al. [11] carried out tool shoulder and probe wear investigations with qualitative and quantitative characterization methods by conducting FSW experiments of workpiece material AA-6060 T66, thereby finding different degrees of tool wear in different process parameters. Furthermore, it was indicated that the wear of the welding tool had a relatively minor impact on the quality of the welded joint. Relative to aluminum alloys, the issue of tool wear is more serious and prominent for FSW of higher hardness and melting point materials, such as steel and titanium, as well as wearable materials such as metal matrix composites [12,13,14,15]. As is known, the welding tool experiences high temperatures, mainly generated by the thermal transfer from the tool–workpiece contact interface during the FSW process. Sato et al. [16] and Colegrove et al. [17] found that the temperature of the contact interface is around 500 °C when welding aluminum alloys, generally exhibiting heat resistance for H13 tools. However, due to the complex thermal–mechanical coupling effects, the welding tool is more likely to undergo multidirectional loads or stresses, resulting in tool failures such as severe deformation and fracture. Su et al. [18] and Huang et al. [19] indicated in their studies that the welding tool may undergo fatigue deformation or fracture due to the alternating loads when welding soft metal materials like aluminum alloys and proposed that compared to wear, fatigue failure of the welding tool should be more worthy of attention.

Recent studies have been focused on the mechanical properties and fatigue life of the welding tool during FSW, employing theoretical calculations, experiments, or numerical simulations. Bradley [20] conducted a rotating–bending fatigue experiment to study the effect of temperature on the fatigue of H13 tool steel, thereby finding that the tool fatigue life dramatically decreases above 500 °C, which indicated that welding temperatures should be controlled below this safe limit. Buchibabu et al. [21] investigated torque and lateral force variations of the tool pin during FSW of 7XXX aluminum alloys using a combination of theoretical modeling and experimental research and analyzing process parameter impacts on tool life by taking the ratio of maximum shear stress to its yield strength as the durability evaluation metric. Moshwan et al. [22] explored the influence of welding parameters on welding tool forces for AA5052 aluminum alloy. Astarita et al. [23] studied the effect of alloy strength and thickness on the load-bearing capacity of the tool pin. Other scholars [24,25,26,27] investigated torque, welding force, and stress distribution on tool pins with different cross-sectional shapes using numerical simulations, revealing insights into the fatigue strength impacted by tool pin configurations. Huang et al. [19] studied the temperature and stress distribution of FSW tools with a high depth-to-width ratio based on the construction of a fluid–solid numerical model, identifying the primary location of tool fracture at the root of the tool pin. In terms of the research on tool fatigue life, Stephen et al. [28] established a model for a temperature–shear force/bending force relationship, by which the distribution of maximum stress acting on the tool pin and the corresponding mechanical properties of the welded joint under specific conditions were observed and compared to optimize the tool life. Based on computational fluid dynamics (CFD) finite element model, Zhang et al. [29,30] utilized the damage accumulation method and KBM multiaxial fatigue formulation to predict the fatigue life of the welding tool for aluminum alloy 6061-T6 under different welding parameters and tool sizes. However, the failure mechanisms, such as the manifestation of fatigue failure and the primary factors in FSW, are subject to further research and confirmation. Furthermore, analyzing thermal stress conditions and exploring stress distribution rules during the FSW process is helpful to understand their influence on tool fatigue life and would be of significant value for the selection and design of welding tools in practice based on scientific principles.

Due to the excellent properties, such as high strength-to-weight ratio, good mechanical properties at low and high temperatures, and high fracture toughness, aluminum alloy 2219 is widely utilized in the aerospace and aviation industries [31,32,33]. In this paper, the welding of AA2219-T4 is taken as a case study to perform experiments on FSW welding and the fracture morphology analysis of the failed tool. The fatigue mechanisms of the tool failure in the FSW process are investigated by constructing an FSW ALE model and a thermal-mechanical stress model in DEFORM software. Meanwhile, the effect of the investigated stress on the fatigue performance of the welding tool is analyzed. The Tool Life module in DEFORM is utilized to predict the fatigue life of the welding tool. Finally, the possibility of a more accurate prediction based on the proposed model in this paper is indicated with more supportive data for the welding tool S-N curve under conditions of more practical fatigue testing experiments.

## 2. Materials and Methods

### 2.1. Tool Material and Geometry

The welding tools used in the experiments conducted for this study are made from H13 tool steel with a hardness of 47-51HRC after heat treatment. Table 1 provides an overview of the physical properties and mechanical performance of H13 tool steel at various temperatures.

**Figure 1:**
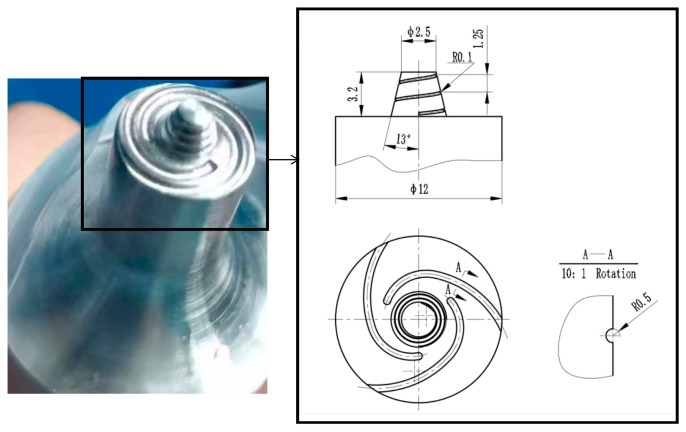
Geometry and dimensions of the tool.

The welding tool is primarily composed of two integral parts, including the shoulder and the pin. To optimize material flow and prevent material overflow beneath the shoulder, a scroll concave groove feature is intentionally incorporated into the end face of the shoulder. The shoulder has a diameter of φ12 mm. The tool pin is conical and includes threads designed to further enhance material flow. It has a length of 1.1 mm and a taper angle of 13°. Figure 1 illustrates the geometry and dimensions of the welding tool.

### 2.2. FSW Experimental Procedure

The welding experiment was conducted using an FSW5020 gantry-type FSW machine. The chosen welding material was AA2219-T4, and its chemical composition and mechanical properties are detailed in Table 2. The specimen dimensions were 300 mm × 130 mm × 3.4 mm. Referencing process design principles and engineering experience [34,35,36], the welding parameters were configured as follows: spindle speed (n) = 1200 rpm, welding speed (f) = 400 mm/min, shoulder penetration depth (p) = 0.4 mm, plunge rate of the welding tool (ν) = 9 mm/min, and the tilt angle of the welding tool was set as 2.5°.

**Figure 2:**
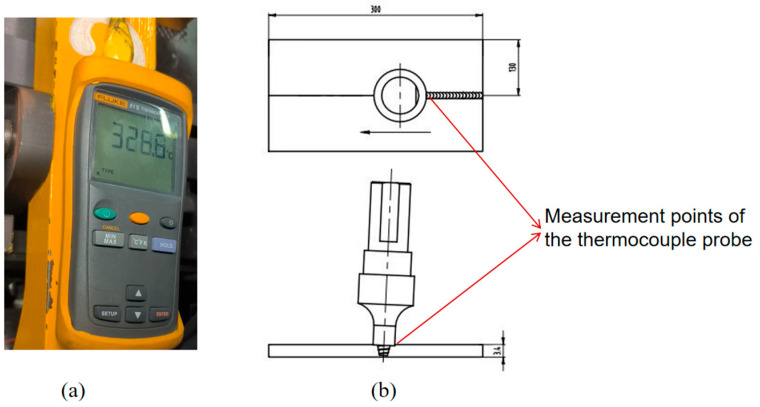
Thermal cycle measurement of the welding tool. (**a**) Handheld contact thermocouple. (**b**) Temperature measurement schematic.

The temperature of the welding tool was monitored using a FLUKE F51-II (Fluke, Everett, WA, USA) handheld contact thermocouple equipped with a temperature measurement range of 0–1372 °C. The thermocouple probe was strategically positioned at the rear of the contact surface between the welding tool and the specimen to dynamically capture temperature variations during the FSW process, as depicted in Figure 2.

**Figure 3:**
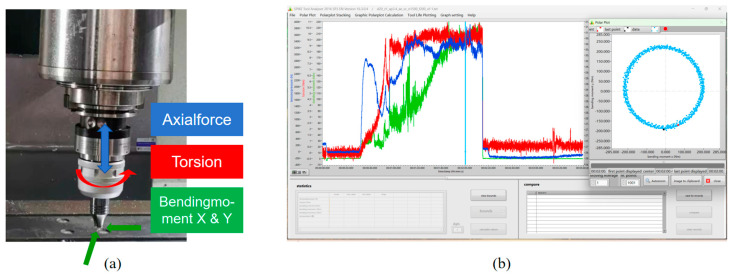
Dynamic measurement by using the Spike system. (**a**) Physical diagram of the measurement system. (**b**) Data transmission and display.

The SPIKE wireless dynamic cutting force measurement system, Spike^®^, was employed to measure the welding force and torque during the FSW process. The measurement sensors were integrated into the tool holder and connected to the spindle of the machine tool with a BT50 interface, while the welding tool was secured at the base. Figure 3a provides a visual representation of the Spike measurement system clamping the welding tool, enabling wireless measurement of axial force, torque, and bending moments in the X and Y directions during FSW. The measurement data are wirelessly transmitted to the receiver at a frequency of 2.5 kHz and displayed and saved in real time in the software on the computer through the receiver connection, correlating with the FSW process detection data, as depicted in Figure 3b.

### 2.3. Numerical Modelling

#### 2.3.1. FSW ALE Model

**Figure 4:**
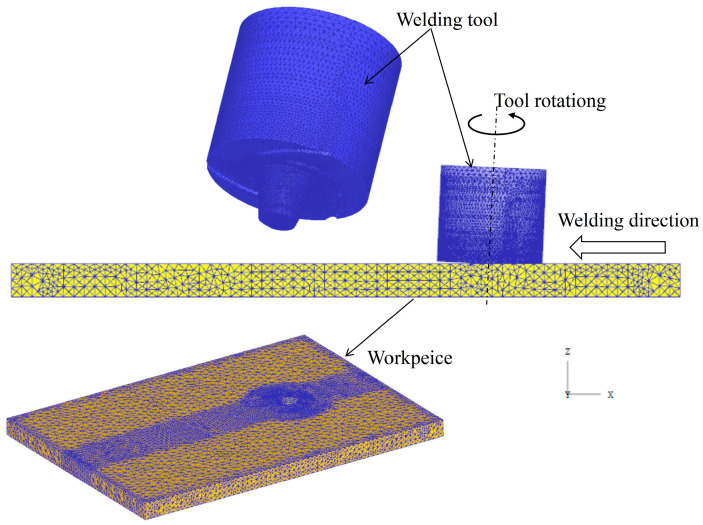
FEM model.

The commercial finite element analysis (FEA) software DEFORM is employed to simulate the FSW process, utilizing the ALE method to establish a three-dimensional thermal-stress coupled model for FSW. The ALE algorithm effectively mitigates excessive grid refinement caused by substantial material deformation during the FSW process, thereby improving computational convergence and speed [37]. In the numerical simulation of the FSW welding process, the finite element method (FEM) model encompasses the welding plate and the tool. The welding plate is modeled as a plastic body, and the tool is modeled as a rigid body, both partitioned with tetrahedral meshes. The method of progressive meshing is utilized to mesh the workpiece and tool. In the contact area between the welding tool and the welding plate, local mesh refinement is applied with the minimum size of the elements specified as 0.4 mm, while mesh elements in other regions are gradually enlarged. Because FSW is a severe plastic deformation process, the absolute mesh setting with an adaptive remeshing method is used in the DEFORM-3D software, which can effectively control the mesh distortion in the simulation. This approach ensures computational accuracy while minimizing computational burden. Accordingly, the total meshed tetrahedral elements of the sheet blank and the tool are 87,394 and 56,583, respectively. For enhanced computational efficiency, the models of the welding plate and tool are simplified. Displacement constraints are separately applied to the bottom and side surfaces of the welding plate, restricting its movement in the Z direction and in the X and Y directions. This reflects the functions of the fixture and backing plate. The FEM is illustrated in Figure 4. To maintain consistency with the FSW welding experiment process, the simulation process is divided into three stages, including tool rotation and downward movement, tool rotation dwell, and tool rotation traverse. The welding tool rotates counterclockwise around the center axis, while the welding direction moves along the negative X-axis. The process parameters align with those used in the FSW experiment. The simulation calculation was finished after about 120 h.

The parameters of the thermal physical properties of the welding plate material 2219-T4 aluminum alloy are defined as constants, as shown in Table 3.

The material constitutive model is described by the tabular format, which can represent any material for which flow stress can be given as a function of strain, strain rate, and temperature [37].

(1) $$\bar{\sigma }=(\bar{\varepsilon },\dot{\bar{\varepsilon }},T)$$

where $\bar{\sigma }$ is flow stress; $\bar{\varepsilon }$ is the effective plastic strain; $\dot{\bar{\varepsilon }}$ is the effective strain rate; and T is temperature.

**Figure 5:**
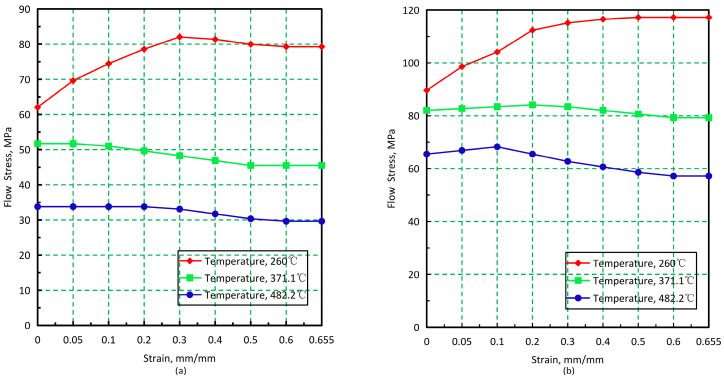
Flow stress at varying temperatures for AL2219 aluminum alloy. (**a**) Strain rate = 0.1 s^−1^; (**b**) Strain rate = 10 s^−1^.

The constitutive relationship curves illustrating the material flow stress at varying temperatures for two different strain rate values of the AL2219 material are depicted in Figure 5.

During the FSW process, materials experience significant frictional heating induced by the welding tool, leading to a predominant thermal plastic deformation state [38]. Consequently, the shear friction model is utilized in the DEFORM software [39], and it is expressed as follows:

(2) $$f_{s}=mk$$

where $f_{s}$ is the interfacial frictional force, and *m* is the shear friction coefficient, with a range of 0 < *m* ≤ 1. In practical welding scenarios, the friction coefficient between the stirring tool and the workpiece is influenced by factors such as temperature, pressure, and velocity at the contact interface, resulting in a complex variation. However, to streamline the model calculations, the value in the FSW friction model is commonly set to 0.7 due to the dry surface of the contacting area between tool and workpiece [37]. Here, *k* represents the shear yield stress of the material.

The tool heat is mainly generated simultaneously by frictional conditions at the tool/workpiece interface and heat transfer from the workpiece. The frictional heating can be calculated from the total heat generation rate $dq_{f}$, which is expressed as follows [40,41]:

(3) $$dq_{f}={(1-\delta )\tau }_{contact}\omega rdA$$

where r is the the distance of one finite element unit to the center of the tool, ω is the tool rotational speed, dA is the unit area, $\tau_{contact}$ is the frictional force, and δ(<1) is a contact state variable, which represents the velocity difference between the contact surface of the tool and the workpiece. 

Considering the heat conduction between the workpiece and the welding tool, as well as the convective heat exchange between the workpiece and the free surface of the welding tool with the surroundings, the thermal transfer conditions in this model are established based on references [39,42] as follows:

1. The convective heat exchange coefficient of the workpiece and the welding tool with the surroundings is set to 20 Wm^−2^K^−1^.
2. The thermal conductivity coefficient at the contact regions between the workpiece and the welding tool is set to 11,000 Wm^−2^K^−1^.

#### 2.3.2. Tool Stress Analysis Model 

It is known that the welding tool is subject to thermal and mechanical loads during FSW. Therefore, the comprehensive stress experienced by the welding tool is the vector sum of thermal stress and mechanical stress, which is indicated in the form of equivalent stress as follows [43,44]:

(4) $$\bar{\sigma }=\frac{1}{\sqrt{2}}\sqrt{{\left(\sigma_{ix}-\sigma_{iy}\right)}^{2}+{\left(\sigma_{iy}-\sigma_{iz}\right)}^{2}+{\left(\sigma_{iz}-\sigma_{ix}\right)}^{2}+6{\left(\tau_{ixy}+\tau_{iyz}+\tau_{izx}\right)}^{2}}$$

where $\sigma_{i}=\sigma_{ij}+\sigma_{ir}$, $\tau_{i}=\tau_{ij}+\tau_{ir}$. j represents the mechanical stress, and r represents the thermal stress.

The vectors of the mechanical stress and thermal stress experienced by the welding tool at a certain time are calculated using Equation (4). According to the principle of vector superposition in elastic bodies, the equivalent stress at that time can be obtained. If any area of the tool is close to or exceeds the yield stress of the tool material, it is indicated that region is at risk of plastic deformation.

On the other hand, the maximum principal stress, including $\sigma_{max}$ or $\sigma_{min}$, indicates which regions of the tool are in a state of tension and which are in a state of compression. If the maximum principal stress is large, it may be a site for fatigue failure initiation.

#### 2.3.3. Tool Fatigue Model

The fatigue model in this study is constructed mainly based on the cyclic stresses of the welding tool. Following the thermal–mechanical coupled simulations of FSW using DEFORM software, the TOOL LIFE module is utilized for the fatigue stress analysis of the welding tool. The welding tool is characterized as an elastic body, in which the velocity of the top surface is set as 0 so that the tool does not fly off into space when the forming loads are applied to it. The cycle function is applied to calculate stress accumulation after 5(n) cycles during the steady-state welding stage. In each cycle, the tool stresses are calculated at multiple steps, based on which the fatigue analysis will be performed.

The Basquin model is employed to construct the high-cycle fatigue life standard S-N curve for the welding tool during the FSW process, forming the basis for fatigue life prediction. The Basquin model is described using Equation (5) as follows:

(5) $$∆\sigma =SRI1{\left(N_{f}\right)}^{b1}$$

where $N_{f}$ refers to the number of cycles to failure, ∆σ denotes the stress amplitude, SRI1 is typically the ultimate tensile strength of the material, and *b_1_* is the fatigue strength exponent which is related to the material composition and microstructure [45].

The alternating cycle stress for fatigue is fundamentally represented as follows:

(6) $$\sigma_{m}=\frac{1}{2}\left(\sigma_{\max}+\sigma_{\min}\right)$$

(7) $$\sigma_{a}=\frac{1}{2}\left(\sigma_{\max}-\sigma_{\min}\right)$$

(8) $$R=\frac{\sigma_{\min}}{\sigma_{\max}}$$

where${\;\sigma }_{m\;}\;$refers to the mean stress, ${\;\sigma }_{a\;}$ refers to the stress amplitude, and R refers to the stress ratio. When R=−1, it represents a symmetric cyclic stress, and when −1<R<1, it represents a nonsymmetric cyclic stress. Under nonsymmetric cyclic stress conditions, the mean stress ${\;\sigma }_{m\;}$ has a significant impact on fatigue life. Typically, when $\sigma_{m\;}>0$, the overall stress state is tensile, resulting in lower fatigue life, whereas when $\sigma_{m\;}<0$, the overall stress state is compressive, resulting in better fatigue life, and a higher ${\;\sigma }_{m\;}$ leads to lower fatigue life.

## 3. Experimental Results

### 3.1. Fracture Failure Analysis of the Tool Fracture Surface

**Figure 6:**
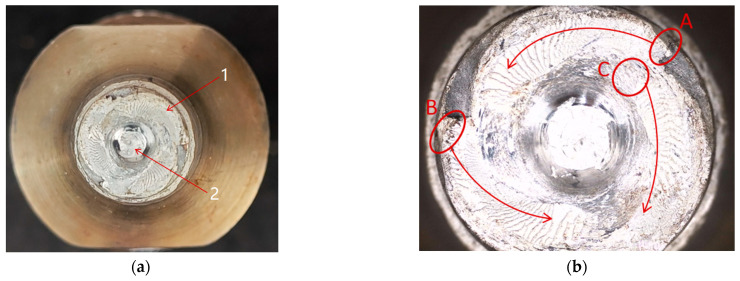
The failed fracture surface of the welding tool. (**a**) The fractured tool. (**b**) Macroscopic morphology of the fractured tool.

The macroscopic morphology of the fractured tool welded to around 1200 m in practice is illustrated in Figure 6a, where one denotes the shoulder end face, and two represents the fracture surface of the tool pin. For a closer examination of the fracture morphology, the failed tool was observed under a microscope. As depicted in Figure 6b, radial shell patterns are evident in both the shoulder surface and the tool pin fracture area, where the shell patterns appear bright white and relatively flat. These areas are inferred as fatigue zones. Additionally, at the center of the tool pin fracture, a fibrous and grayish-white appearance was observed, indicating the final fracture area.

Notably, the shell patterns converge at the position circled in red in Figure 6b, with positions A and B situated on the outer surface and position C at the junction of the internal structure. This observation reveals multisource fatigue characteristics, and the direction of fatigue crack propagation varies.

**Figure 7:**
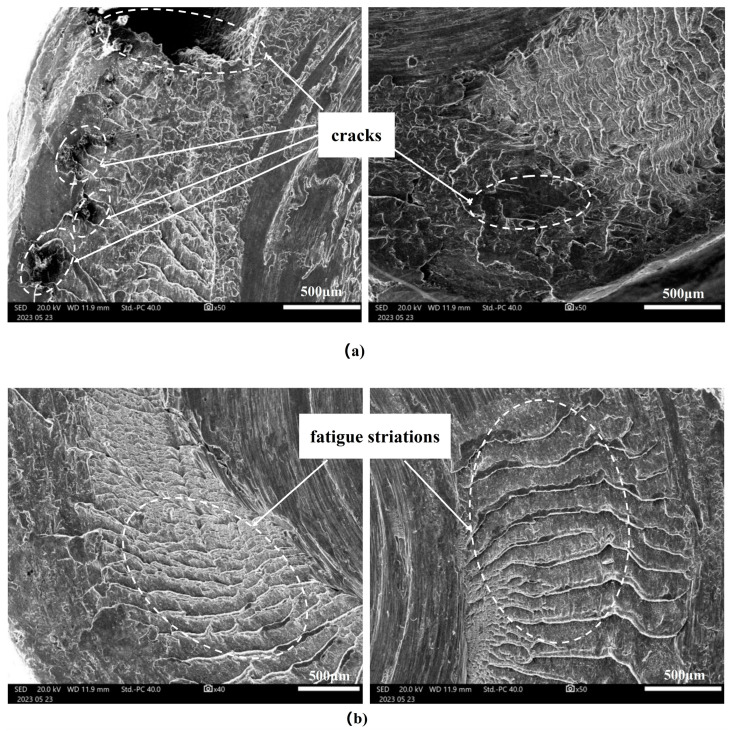
The microscopic morphology of the failed fracture tool shoulder. (**a**) Crack origin areas. (**b**) Crack propagation zones.

In Figure 7, the microscopic morphology of the tool shoulder is depicted through scanning electron microscope (SEM) observation. Cracks are discernible, originating from localized regions on the outer side of the shoulder, as shown in Figure 7a. No apparent mechanical damage or metallurgical defects, such as inclusions, are visible on the surface. In proximity to the crack origin, a stepped fracture pattern parallel to the axial direction is evident, as shown in Figure 7b. This observation suggests that the fatigue failure of the shoulder primarily results from torsional fatigue induced by shear stress.

**Figure 8:**
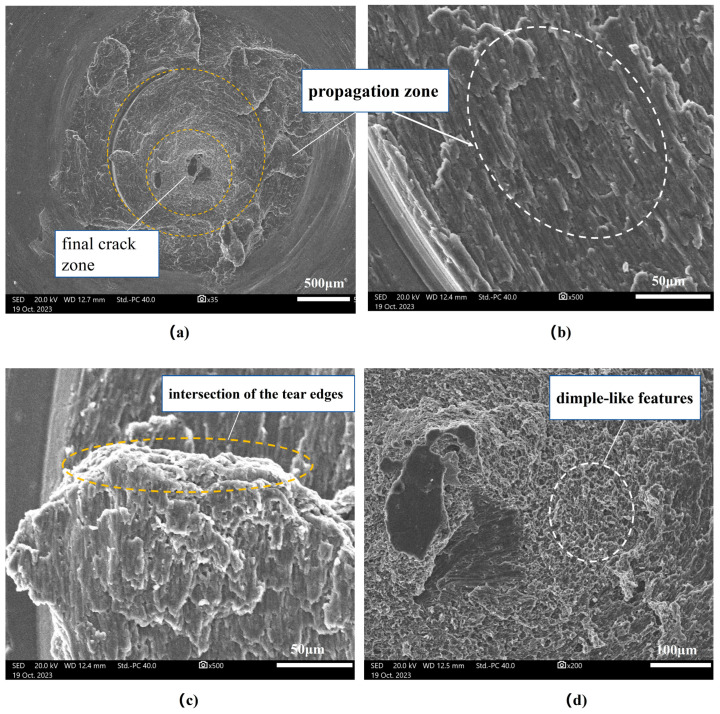
The microscopic morphology of the fracture tool pin. (**a**) At a magnification of 35 times. (**b**) Outer area of the propagation zone at a magnification of 500 times. (**c**) Inner area of the propagation zone at a magnification of 500 times. (**d**) Center area of final crack zone at a magnification of 200 times.

The microscopic morphology of the fracture surface of the tool pin, observed under different magnifications using SEM, is presented in Figure 8. At 35 times magnification (Figure 8a), the outer periphery of the fracture appears relatively flat. Subsequently, at 500 times magnification (Figure 8b), a cleavage morphology is evident, with fine fatigue striations observed on the cleavage surface, forming a ring pattern. This observation indicates that the crack originated from the outer periphery and propagated inward. In Figure 8c, distinct divergent tearing edges are visible in the propagation zone at 500 times magnification. These tearing edges converge at the center of the fracture, showcasing multiple fatigue sources, as indicated by the intersection of the tear edges on the propagation surface. Figure 8d illustrates the fibrous core at the center of the fracture, displaying a significant proportion of fibrous regions. Upon further magnification, the fibrous core exhibits dimple-like features, which are indicative of overload fracture characteristics. This suggests the final fracture zone, implying that the main cause of failure is a fatigue fracture due to significant bending stress experienced during service.

### 3.2. FSW Simulation Model Verification

**Figure 9:**
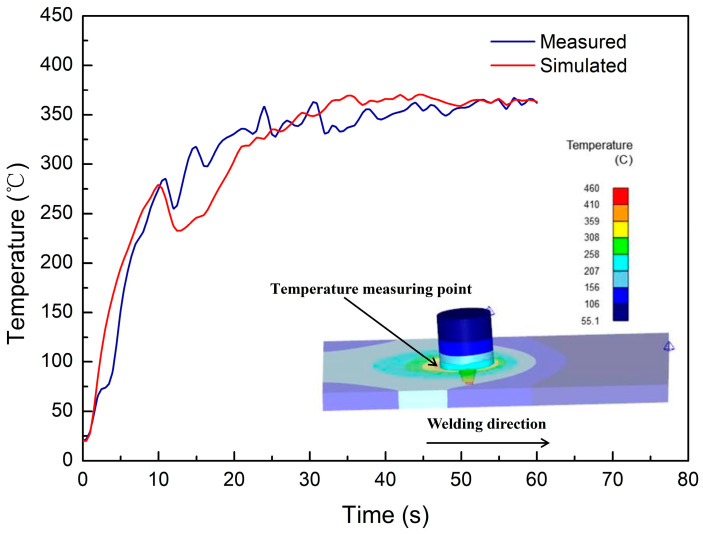
Comparison between simulated and measured temperature of the tool.

The comparison between the measured temperature with the handheld thermocouple and the simulated temperature under the same process conditions is depicted in Figure 9. Throughout the FSW process, both temperature variation trends of the tool align consistently across the plunge, dwell, and steady-state welding stages.

Commencing from the initial contact between the tool pin and the workpiece, the temperature rapidly rises to around 270 °C as the tool shoulder plunges into the material to a certain depth due to the generation of frictional heat and the effect of heat conduction. During the dwell stage, the temperature slightly decreases to approximately 250 °C. Subsequently, as the tool moves along the weld seam, the temperature gradually increases to around 350 °C. Before the completion of welding, the temperature remains stable within a small fluctuation range of 350 °C to 370 °C. Due to the use of handheld thermocouples, which cannot ensure constant contact between the probe and the measuring position of the tool head, the measured temperatures exhibit relatively slight fluctuations compared to the temperature curve of the numerical simulation. However, as described above, the overall trends are consistent, and the maximum error during the steady-state welding stage is below 8%. It is notable that there was a slight decrease in the temperature variation during the simulation of dwelling, which may be inferred that the stopping downward movement of the welding tool reduces the forging forces, resulting in a decrease in friction stress and, to a certain extent, a reduction in the generation of frictional heat.

**Figure 10:**
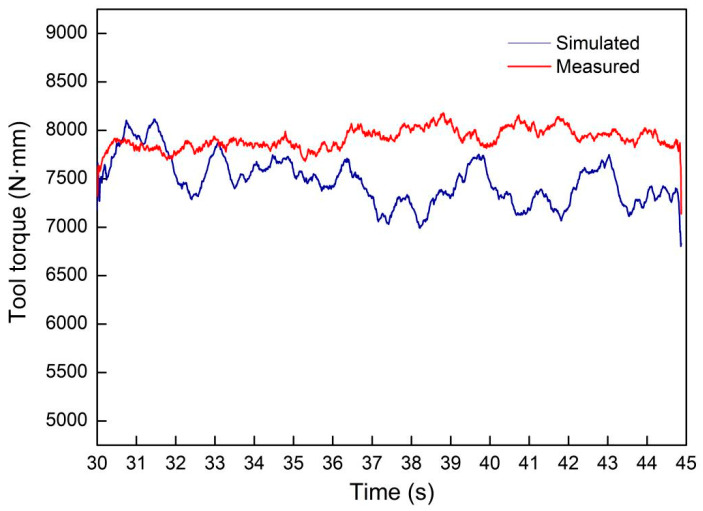
Comparison between simulated and measured torque of the welding tool.

The comparison between the experimental measured and simulation results of the welding tool torque during the FSW steady-state welding stage is shown in Figure 10. It can be seen that the simulated values are lower than the experimental values, with a maximum deviation of 12%, and that there are some fluctuations in the simulated values, which may be attributed to the local data distortion caused by remeshing during simulation calculations. However, the overall trend remains consistent as seen.

**Figure 11:**
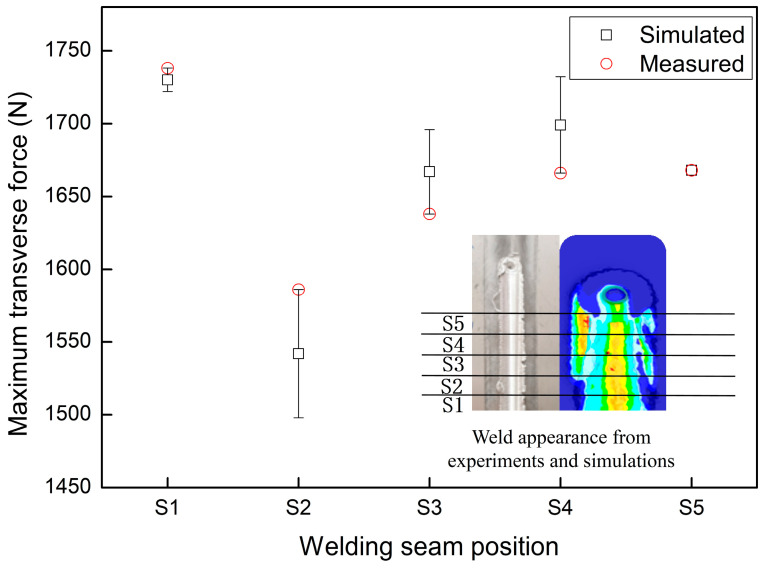
Comparison between simulated and measured transverse forces of the welding tool at different positions along the weld seam.

The experimental and simulated results of the maximum transverse force values, measured at intervals of 3 mm during the steady-state welding stage, are depicted in Figure 11. It can be observed that the maximum error bar is within 3% between the experimental and simulated results, and the variation trend remains consistent. However, the standard deviation data for the traverse force was rarely reported for FSW of AA2219 alloys [18,22,23,25]. The indicated error bar of 3% is acceptable compared with the relevant research reports.

## 4. Simulation Results and Discussions

The comparisons between the experimental and simulation results of temperature, torque, lateral force, etc., under the same FSW process conditions generally exhibit good agreements, suggesting that the established simulation model is relatively accurate and reliable. Building on this, numerical simulations are applied to analyze the temperature field, stress field, and other variations of the welding tool under the thermal–mechanical coupling effect, with the goal of studying the fatigue failure mechanism and tool life.

While the welding tool experiences maximum stress when the tool pin plunges into the workpiece, the duration of this pressure is relatively short compared to the entire FSW welding process. Therefore, the stress during this process is not considered the main cause of the fatigue phenomenon in the welding tool [23]. As this study primarily focuses on the fatigue performance of the welding tool, physical quantities, such as the temperature field and stress field of the tool during the steady-state welding stage, are emphasized in the analysis.

### 4.1. Temperature Field of the Welding Tool

**Figure 12:**
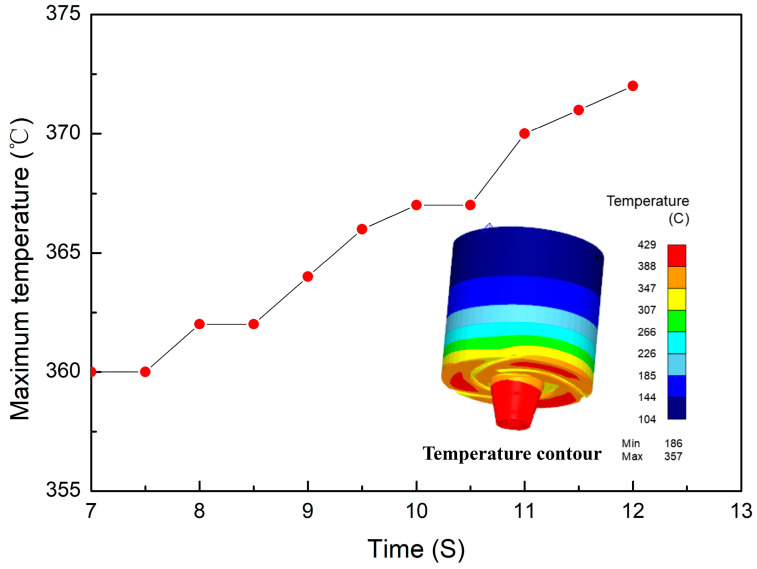
The temperature contour and the maximum temperature curve of the welding tool during the steady state welding stage.

The simulated temperature contour and the maximum temperature curve of the welding tool during the steady-state welding stage are illustrated in Figure 12. It is evident that the maximum temperature is situated at the bottom of the tool pin. As the tool progresses along the weld seam, its temperature remains within the range of 360 °C to 375 °C with slight fluctuations. Therefore, it can be inferred that minimal thermal stresses were generated in the welding tool during the steady-state welding stage when welding aluminum alloys, indicating favorable thermal fatigue performance.

**Figure 13:**
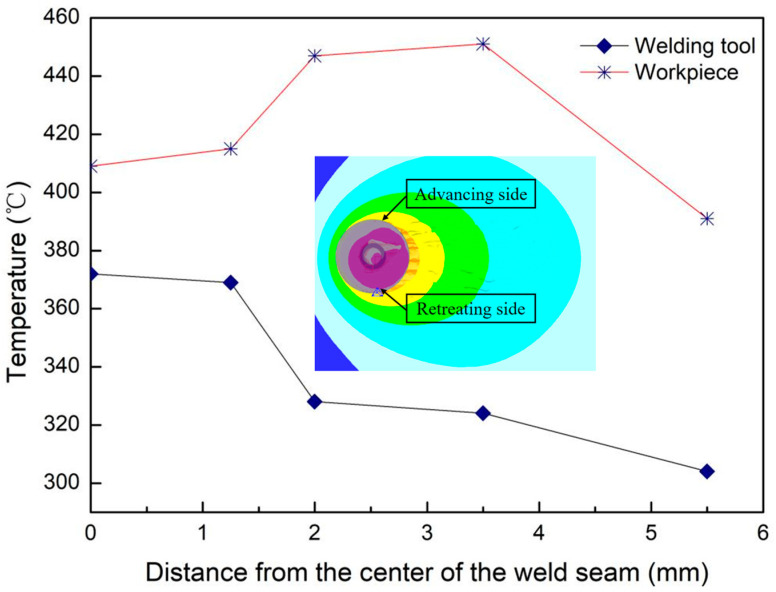
The temperature distribution along the radial distance from the center of the weld on the retreating side.

Figure 13 displays the temperature distributions of the welding tool and the workpiece along the radial distance from the center of the weld on the retreating side. Observing from the center of the weld to the edge of the shoulder, the temperature of the welding tool decreases from 372 °C to 304 °C. Correspondingly, the welding temperature of the workpiece increases from 409 °C to 451 °C at the position where it reaches the maximum value, and then decreases to 391 °C at the contact position with the edge of the tool shoulder. The temperature of the tool is lower than the workpiece welding temperature, primarily due to the heat generated by the friction between the tool and the workpiece. The contact area between the shoulder and the workpiece is the largest, resulting in the highest heat generation in this area, leading to the highest temperature of the workpiece. However, rapid heat dissipation from the edge of the shoulder to the surrounding material and the external environment causes a decrease in the temperature of the workpiece at this position. Because heat is transferred through contact between the tool and the workpiece, the temperature of the welding head is lower than the welding temperature of the workpiece. On the other hand, the area at the center of the welding tool experiences the fastest generation and transfer of frictional heat, resulting in the highest temperature in this region. As heat is transferred from the welding tool surface to the surroundings, the temperature in the shoulder area gradually decreases outward. Notably, during the steady-state welding stage, the temperature of the stirring head remains below 400 °C, within the tempering temperature of 500 °C for H13 mold steel. This temperature range does not significantly affect surface hardness or pose oxidative wear issues [46,47].

### 4.2. Material Flow Behavior

**Figure 14:**
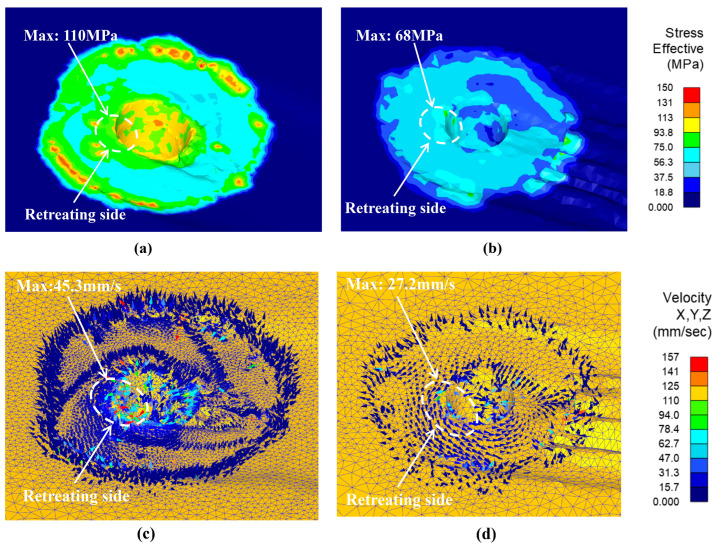
The material flow behavior of the welding tools with thread structure and smooth face structure. (**a**) Flow stress of the thread structure tool. (**b**) Flow stress of the smooth face structure tool. (**c**) Flow velocity of the thread structure tool. (**d**) Flow velocity of the smooth face structure tool.

It is known that the softening of the thermally plasticized materials in the contact areas of the tool and workpiece has a significant impact on the forces of the welding tool during the FSW process, as well as the welding formation [48,49,50]. Figure 14 shows the material flow behavior of the welding tools with the structure designed in this study and without the thread under the same welding parameters. Through comparison, it is found that the distribution of material flow stress is more uniform with the threaded structure welding tool (as shown in Figure 14a,b), and material flow is faster (as seen in Figure 14c,d), especially in the contact area between the tool pin and the workpiece. Furthermore, it is indicated that the regions with higher flow behavior are located on the retreating side of the tool, where the maximum flow stresses and velocity are found, which is consistent with the results of most scholars’ research. Therefore, the welding tool with the thread structure used in this study has significant advantages in promoting material flow.

### 4.3. Stress Analysis of the Welding Tool

**Figure 15:**
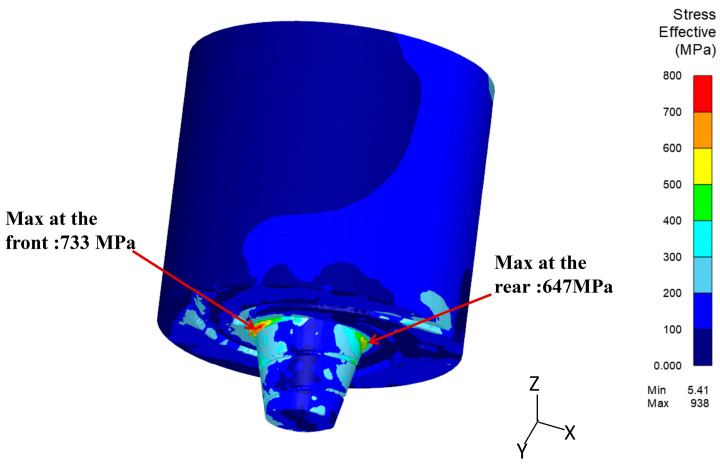
Equivalent stress distribution of the welding tool at step 2128.

Figure 15 illustrates the distribution of equivalent stress during the steady-state welding phase. High stress concentrations are primarily observed in the front and rear of the pin root. The maximum stress in the front region reaches approximately 733 MPa, while the stress in the rear region is approximately 647 MPa. It is noteworthy that the maximum stress is lower than the yield stress of H13 mold steel under equivalent temperatures, which is approximately 1100 MPa.

While the stress distribution is not sufficient to cause ductile fracture failure in the tool pin, the regions mentioned above still have the possibility of experiencing deformation or fracture failure. This observation is consistent with the location of the fractured tool shown in Figure 6.

**Figure 16:**
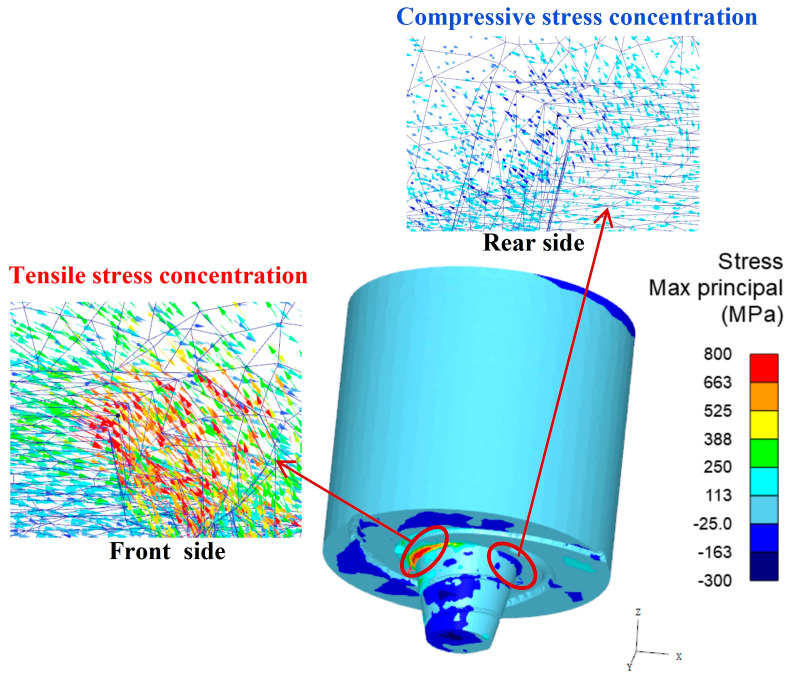
The maximum principal stress distribution of the welding tool.

Figure 16 illustrates the distribution of the maximum principal stress in the welding tool. It can be observed that the region in front of the pin root is the area of maximum tensile stress concentration, while the region behind the pin root experiences the maximum compressive stress concentration.

**Figure 17:**
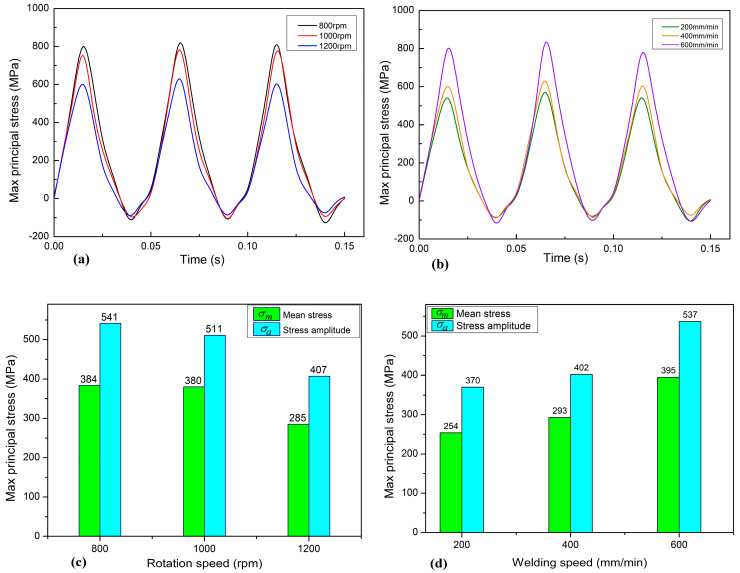
The variation curve of the maximum principal stress at the root of the tool pin. (**a**,**c**) Under the process parameter conditions of rotation speed. (**b**,**d**) Under the process parameter conditions of welding speed.

The variation curve of the maximum principal stress at the root of the tool pin during the steady-state welding is depicted in Figure 17. As shown in Figure 17a,b, it is evident that the stress at the root of the tool pin undergoes periodic and alternating changes between tension in the front and compression in the rear. Moreover, the maximum tensile stress is greater than the maximum compressive stress, indicating that under this alternating mechanical stress, fatigue failure is prone to occur at the root of the tool pin, with the maximum stress at the corresponding position falling within the yield stress range. This further confirms that the fatigue form is mechanical high-cycle fatigue.

In Figure 17c, it can be observed that under the process parameter conditions of a welding speed of 400 mm/min and rotation speeds of 800 rpm, 1000 rpm, and 1200 rpm, the corresponding average stresses at the root of the tool pin are 384 MPa, 380 MPa, and 285 MPa, respectively, and the corresponding stress amplitudes are 541 MPa, 511 MPa, and 407 MPa, respectively. Increasing the rotation speed reduces the average stress and stress amplitude of the tool pin, thereby increasing the fatigue life of the welding tool. Under a rotation speed of 1200 rpm, the reduction in both average stress and stress amplitude is more significant compared to the other conditions, which is more favorable for improving the fatigue life of the welding tool.

In Figure 17d, under a rotation speed of 1200 rpm and welding speeds of 200 mm/min, 400 mm/min, and 600 mm/min, the corresponding average stresses at the root of the tool pin are 254 MPa, 293 MPa, and 395 MPa, respectively, and the corresponding stress amplitudes are 370 MPa, 402 MPa, and 537 MPa, respectively. With the increase in welding speed, the average stress and stress amplitude of the tool pin increase, thereby reducing the fatigue life of the welding tool. Under a welding speed of 600 mm/min, the increase in both average stress and stress amplitude is more significant, negatively impacting the fatigue life of the welding tool.

### 4.4. Fatigue Life Prediction of the Welding Tool

It is known that the welding tool experiences multiaxial stresses, such as tension, compression, torsion, and bending, during steady-state welding in FSW, by which multiaxial fatigue is likely to be induced. Considering the simplification of the calculation model, the maximum principal stress of the tool is taken as the fatigue analysis stress for the S-N curve.

The stress analysis of the welding tool from Figure 17 reveals that all average stresses in the tool pin root are greater than zero. Accordingly, its corresponding S-N curve is lower than the standard S-N curve. Although the stress results indicate that the tool pin is subjected to asymmetric cyclic stress, the overall trend remains consistent between the corrected and standard S-N curves [20,51].

**Figure 18:**
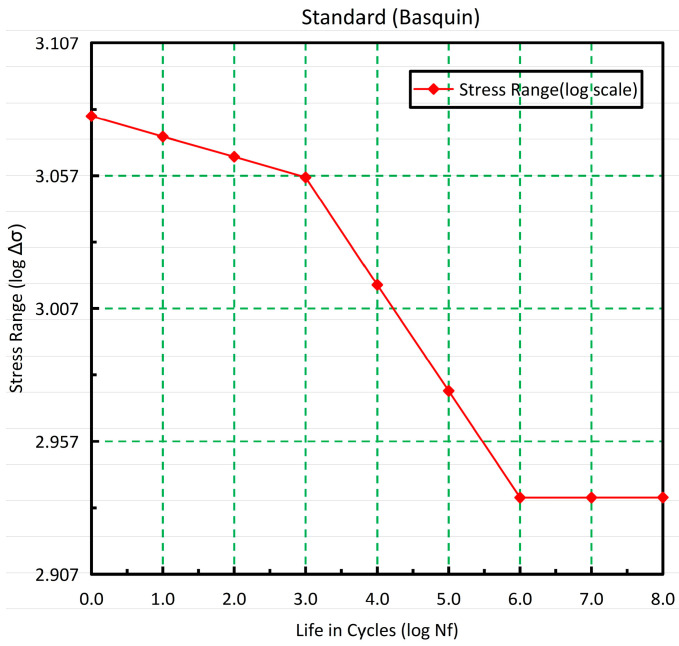
The standard S-N curve for H13 steel.

Because obtaining the actual S-N curve of the welding tool through experiments is challenging under the present experimental conditions, the standard S-N curve has been adopted for the fatigue life analysis based on stress analysis in this study. As shown in Figure 18, the standard S-N curve of H13 steel constructed by the Basquin model was used for the fatigue life analysis and prediction. In this model, the parameter settings for the S-N curve are as follows: SRI1 = 1200, b_1_ = −0.04, N_f1_ = 1000, N_f2_ = 1,000,000.

**Figure 19:**
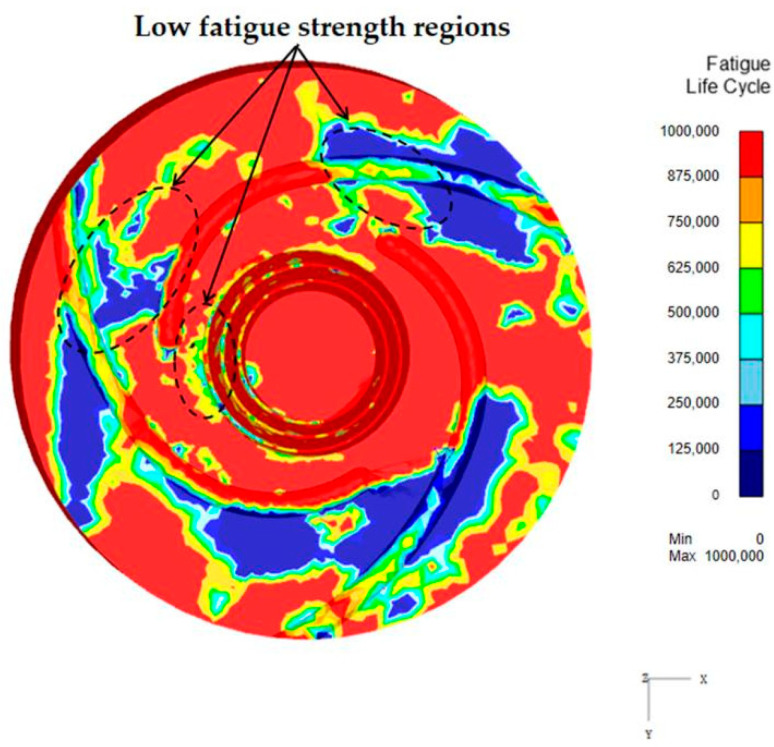
The simulation results of the fatigue life prediction for the welding tool.

The predicted fatigue life of the welding tool with a rotation speed of 1200 rpm and a welding speed of 400 mm/min, obtained using the Tool Life module in DEFORM software, is illustrated in Figure 19. The fatigue lifecycle here represents the total service life under the same process conditions. The larger is the fatigue life-cycle, the longer is the fatigue life. As a result, it is apparent that the areas with lower fatigue strength on the welding tool are primarily situated at the root of the tool pin and in the local area of the scroll concave groove on the shoulder face. This is consistent with the experimental findings obtained from the morphology analysis of the tool fracture surface, as shown in Figure 6 and Figure 7.

**Figure 20:**
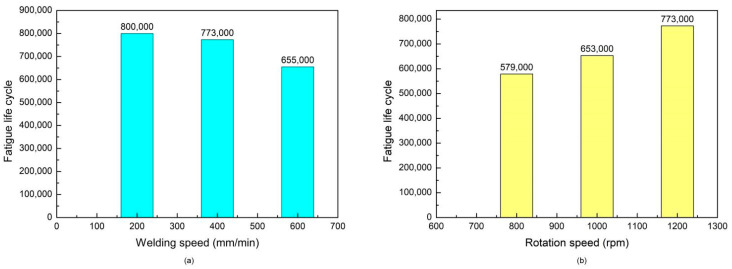
The fatigue life prediction of the tool under different welding process parameters. (**a**) Under the same rotation speed of 1200 rpm and various welding speeds of 200 mm/min, 400 mm/min, and 600 mm/min. (**b**) Under the same welding speed of 400 mm/min and various rotation speeds of 800 rpm, 1000 rpm, and 1200 rpm.

The fatigue lives of the tool under different welding process parameters, as predicted by this model, are presented in Figure 20. Figure 20a shows the fatigue life variation under the same rotation speed of 1200 rpm and various welding speeds of 200 mm/min, 400 mm/min, and 600 mm/min, and Figure 20b illustrates the fatigue life variation under the same welding speed of 400 mm/min and various rotation speeds of 800 rpm, 1000 rpm, and 1200 rpm. It can be indicated that reducing the welding speed and increasing the rotation speed can enhance the fatigue life of the welding tool to some extent.

### 4.5. Further Analysis of Fatigue Failure Mechanism of Welding Tool

**Figure 21:**
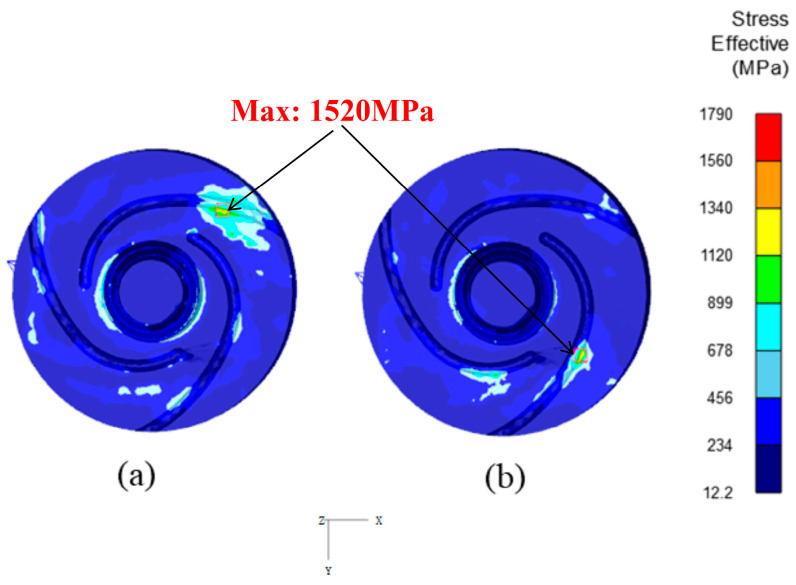
The distribution of equivalent stress on the welding tool during the steady-state welding phase. (**a**) At the step of 2090. (**b**) At the step of 2130.

Upon closer examination of Figure 18, it is evident that the fatigue life of the tool in the local area of the scroll concave groove on the end face of the tool shoulder is lower compared to the root of the tool pin. In Figure 21, the distribution of equivalent stress on the welding tool during the steady-state welding is illustrated. It can be observed that, in addition to the root of the tool pin, the equivalent stress borne by the local position of the scroll concave groove on the end face of the tool shoulder reaches up to 1520 MPa, exceeding the yield strength and even the tensile strength of H13 steel material. This may lead to the initiation of a crack in this area, primarily because of the stress concentration at the junction of the scroll concave groove and the tool shoulder end face, resulting in increased local stress and subsequent brittle fracture.

**Figure 22:**
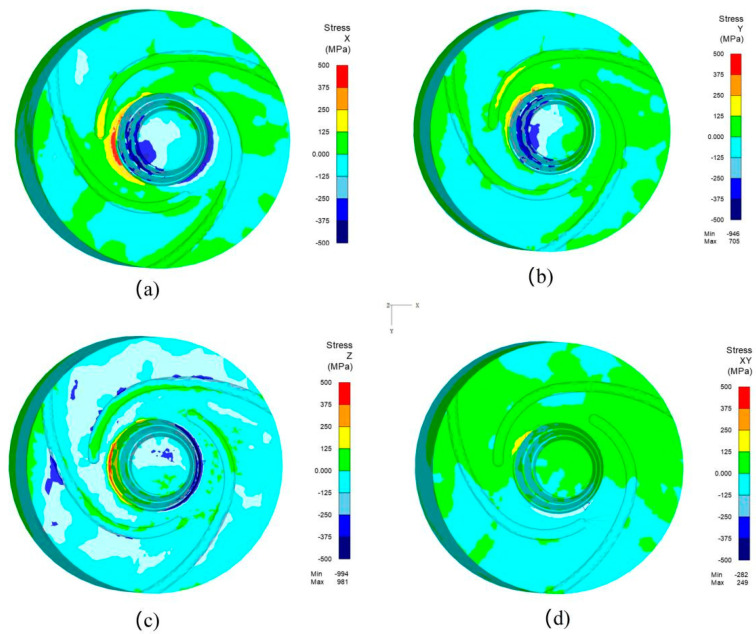
The stress distributions of the welding tool in different directions. (**a**) Stress in the X direction. (**b**) Stress in the Y direction. (**c**) Stress in the Z direction. (**d**) Stress in the XY plane.

The stress distributions of the tool pin in the X, Y, and Z directions, as well as in the XY plane, were obtained using numerical simulation, in which the rotation speed is 1200 rpm and the welding speed is 400 mm/min, as shown in Figure 22. It can be observed that the positive and negative stresses are alternately distributed at the root of the tool pin in different directions, indicating that the root of the tool pin experiences multidirectional stresses, such as bending, tension, compression, and torsion, during the welding process, exhibiting complex fatigue behavior. This result further explains the presence of multiple crack sources observed in the fracture surface of the tool pin, as shown in Figure 6.

The asymmetric cyclic stress values at the root of the tool pin in different directions are presented in Table 4. Upon comparison, it is revealed that the stress amplitude values at the root of the tool pin in the X and Z directions are larger. However, the average stress in the X direction is greater than zero, while in the Z direction, it is less than zero, indicating that the root of the tool pin is under tension in the X direction and compression in the Z direction. Thus, the stress in the X direction has a greater impact on the fatigue life. Although the stress amplitude in the Y direction is relatively smaller compared to the X direction, the average stress is much greater than zero, indicating that tension in the Y direction is unfavorable for fatigue life, as well. In contrast, the XY shear stress is minimal, with an average stress of zero, resulting in a lesser impact on the fatigue life. From the analysis above, it can be inferred that the fatigue life of the tool pin at the root is mainly influenced by bending alternating cyclic stress during FSW welding, which is consistent with the observed morphology of the tool pin fracture in Figure 8.

### 4.6. Impact on the the Technological Design

**Figure 23:**
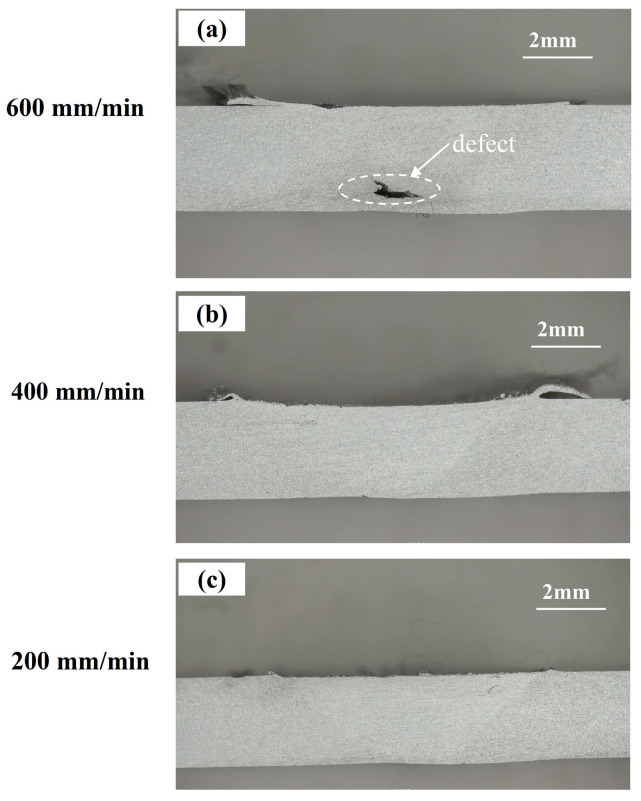
The cross-sectional morphologies of the welds in different welding speeds. (**a**) 600 mm/min. (**b**) 400 mm/min. (**c**) 200 mm/min.

Figure 23 illustrates the cross-sectional morphologies obtained using low-magnification metallographic experiments under the same process parameters as in Figure 20a. It can be seen that void defects appear in the weld under the welding speed of 600 mm/min (shown in Figure 23a). However, no macroscopic defects are found in either weld with welding speeds of 400 mm/min and 200 mm/min (shown in Figure 23b,c). The results indicate that when the rotation speed is too high, weld defects are more likely to occur due to insufficient material flow, and the welding tool is more prone to failure as a result of the increase of fatigue stress. However, through further comparative analysis of Figure 20a and Figure 23b,c, it can be inferred that whether the welding speed is 400 mm/min or 200 mm/min, both the weld qualities and the fatigue performances of the welding tool can be more satisfactory. Taking into account production efficiency in the manufacturing process, as well, it can be inferred that a welding speed of 400 mm/min and a rotational speed of 1200 rpm may be the most suitable process parameters. Therefore, balancing the technological design between quality and efficiency should be considered before the practical manufacturing process.

## 5. Conclusions

The conclusions drawn from the study on the fatigue properties of a welding tool during the FSW welding of aluminum alloys are summarized as follows:

1. The primary failure mode of the welding tool in the FSW of low-melting point alloys, such as aluminum alloys, is fatigue fracture. This mainly occurs at the root of the tool pin. The maximum temperature fluctuations of the welding tool are within a small range of 360 °C to 375 °C, ruling out thermal fatigue as a significant mechanism.
2. Numerical simulations indicate that the maximum equivalent stress is concentrated at the root of the tool pin and at local positions of the tool shoulder with scroll concave grooves. This aligns with experimental results of fracture surface analysis, highlighting that structural changes can lead to stress concentration and crack initiation.
3. At the root of the tool pin, the maximum principal stress alternates cyclically between tension and compression, with the maximum tensile stress exceeding the maximum compressive stress. The fatigue mechanism primarily involves high-cycle mechanical fatigue due to alternating mechanical tensile and compressive stresses. Multidirectional stresses, such as bending, tension, compression, and torsion, contribute to compound mechanical fatigue, with cyclic bending stress having the most significant influence.
4. Increasing rotational speed reduces average stress and stress amplitude at the root of the tool pin, enhancing the fatigue life of the welding tool. Conversely, increasing welding speed results in higher average stress and stress amplitude, thereby reducing the fatigue life. A rotational speed of 1200 rpm is found to be more favorable for enhancing fatigue life, while a welding speed of 600 mm/min has a significant negative impact.
5. The fatigue life prediction, which was conducted using the Tool Life module in DEFORM, aligns with experimental results to some extent, demonstrating the reliability of the prediction model. However, due to the limitation of collecting experimental data for the S-N curve of the welding tool, the accuracy of the fatigue life prediction model still needs further research and improvement in future work.

The study emphasizes the feasibility of the research methodology through experiments and numerical simulations to evaluate the fatigue performance of welding tools. Future research directions include exploring the fatigue performance of welding tools with different structures, welding plates of various thicknesses, and utilizing aluminum alloy materials with different levels of performance.

## Figures and Tables

**Table 1:** Physical properties and mechanical characteristics of H13 at different temperatures.

Thermal Expansion Coefficient (10^−6^ K)		Thermal Conductivity (W/(m∙K))		Yield Strength (MPa)		Hardness (HRC)	
20~100 °C	10.4	215 °C	28.6	20~250 °C	1455	20 °C	50.2
100~200 °C	11.5	350 °C	28.4	250~425 °C	1180	400 °C	48.7
200~425 °C	12.2	475 °C	28.4	425~600 °C	820	540 °C	45.8
425~540 °C	12.4	605 °C	28.7	600~650 °C	380	600 °C	29.0
540~650 °C	13.1					650 °C	22.7

**Table 2:** Chemical composition and mechanical properties of AA2219-T4.

Chemical Composition (wt%)									Mechanical Properties	
Al	Si	Mn	Mg	Fe	Zr	CU	Ti	V	Tensile Strength (MPa)	Extension Rate (%)
REM	0.08	0.26	0.005	0.12	0.15	6.18	0.42	0.07	315	8

**Table 3:** Physical properties of 2219-T4 aluminum alloy.

Young’s Modulus (GPa)	Density (kg/m²)	Poisson’s Ratio ν	Thermal Conductivity (W/m/°C)	Heat Capacity (J/kg/°C)
68.9	2840	0.33	180	2.43

**Table 4:** Fatigue life prediction in different welding parameters.

Direction	$\mathrm{Max}\;\mathrm{Stress}\;\;(\mathrm{MPa})\;\sigma_{\max}$	Min Stress (MPa)	$\sigma_{a}$ (MPa)	$\sigma_{m}$ (MPa)
X	695	−509	602	93
Y	395	−241	318	154
Z	569	−671	620	−51
XY shear plane	171	−171	171	0

## Data Availability

The data presented in this study are available on request from the corresponding authors.
